# Configurational pathways to effective rural older adult sports participation: a necessity and sufficiency analysis using NCA and QCA

**DOI:** 10.3389/fpubh.2025.1695787

**Published:** 2025-10-27

**Authors:** Huan Feng, YanJin Li, XiaoYi Wang, QingChuan Wang, ZhiHua Wang

**Affiliations:** ^1^College of Physical Education, Sichuan University, Chengdu, China; ^2^College of Physical Education, Jiangxi Normal University, Nanchang, China; ^3^Competitive Sports College, Shanghai University of Sport, Shanghai, China; ^4^College of Sports, Chengdu Sport University, Chengdu, China

**Keywords:** rural older adult(s), sports participation, configurational analysis, necessary condition analysis, qualitative comparative analysis, public service delivery, rural governance

## Abstract

**Background:**

Rural older adult populations face significant disparities in sports participation compared to urban areas. Traditional linear analytical approaches often fail to capture the complex configurational nature of effective public service delivery in rural contexts, necessitating sophisticated methodological approaches that accommodate multiple pathways to effectiveness.

**Methods:**

This study employed Necessary Condition Analysis (NCA) and Qualitative Comparative Analysis (QCA) to examine village-level conditions promoting effective rural older adult sports participation in China. Data were collected from 156 villages across three regional contexts, using a multi-stage stratified sampling design. Seven conditions were analyzed: Leadership Support (LD), Planning Systems (PS), Specialized Personnel (PN), Funding Allocation (FD), Facility Infrastructure (FT), Organizational Capacity (OG), and Activity Implementation (AT). NCA identified necessary conditions using ceiling envelopment with free disposal hull (CE-FDH) and ceiling regression with free disposal hull (CR-FDH) techniques, while crisp-set QCA revealed sufficient configurational pathways.

**Results:**

NCA revealed no universally necessary conditions, indicating rural communities can achieve success through multiple alternative pathways without specific prerequisites. QCA identified six distinct sufficient configurations with solution consistency of 0.886 and coverage of 0.43, demonstrating equifinality in rural governance. Specialized Personnel emerged as the only condition present across all pathways (100% frequency), while Leadership Support appeared in five of six solutions (83.3% frequency). Configurations ranged from governance-focused approaches that emphasized leadership coordination to resource-intensive models that integrated formal planning and funding. One pathway achieved effectiveness without traditional Leadership Support, suggesting institutional systems can substitute for individual leadership commitment.

**Conclusion:**

The absence of necessary conditions challenges policy frameworks assuming uniform implementation requirements and supports flexible, context-responsive governance models. Multiple sufficient pathways indicate that effective rural development strategies should accommodate diverse approaches rather than relying on prescriptive solutions. Specialized Personnel represent a fundamental requirement across all configurations, while frequent “don't care” conditions reveal significant substitutability among governance arrangements. These findings contribute to the application of configurational methodology in public administration, providing practical guidance for adaptive rural development policies tailored to local conditions.

## 1 Introduction

Global demographic shifts represent one of the most transformative forces of the 21st century, with population aging emerging as a defining characteristic of contemporary societies (1). By 2050, the global population aged 60 and above is projected to reach 2.1 billion, creating unprecedented challenges for public health systems, particularly in rural areas where outmigration of younger populations has accelerated aging processes (2, 3). Among diverse interventions for promoting healthy aging, sports participation offers unique benefits beyond general physical activity, providing structured opportunities for social interaction and community engagement that are especially valuable for older adult populations (4, 5). Research consistently demonstrates that older adult sports participation reduces cardiovascular disease risk, enhances cognitive function, and improves overall quality of life (6). However, significant disparities persist between urban and rural contexts, with rural communities facing complex challenges, including limited infrastructure, resource scarcity, and fragmented governance structures that traditional linear analytical approaches have struggled to adequately capture (7, 8).

Traditional approaches to understanding sports participation have predominantly relied on regression-based analyses that assume uniform causal effects across contexts. However, the complex nature of rural governance systems suggests that effective older adult sports participation emerges through configurations of local conditions rather than simple additive relationships (9). Recent methodological advances in configurational comparative analysis offer promising alternatives for understanding such complex social outcomes. Qualitative Comparative Analysis (QCA) enables researchers to identify multiple pathways to the same outcome (equifinality) while recognizing conjunctural causation, where conditions have different effects depending on context (10, 11). Complementing QCA, Necessary Condition Analysis (NCA) provides crucial insights into which conditions are indispensable for achieving desired outcomes (12, 13). The integration of these approaches allows for comprehensive analysis of both necessary conditions that must be present and sufficient pathways that can be pursued, aligning with the complex realities of rural governance where certain foundational elements may be universally required while communities achieve success through diverse combinations of strategies (14).

This study addresses these methodological and substantive challenges by examining village-level conditions that promote effective older adult sports participation in rural China, where rapid aging and evolving governance structures create both opportunities and constraints for public service delivery (15, 16). Drawing on public service supply theory and utilizing data from village administrators across diverse rural contexts, we employ both NCA and QCA to uncover the necessity and sufficiency relationships underlying successful older adult sports participation. Our analysis reveals that effective participation emerges through distinct configurational pathways, rather than uniform policy interventions. Certain conditions prove necessary across contexts, while sufficient conditions manifest through multiple alternative combinations. These findings contribute to three interconnected literature streams: healthy aging in rural contexts, the application of configurational methods to public service delivery, and the understanding of complex causation in social policy outcomes (17, 18). By demonstrating how necessity and sufficiency analyses complement each other, this research advances both theoretical knowledge and methodological practice in the study of rural governance and public service provision.

## 2 Literature review and empirical strategy design

### 2.1 Rural–urban disparities in older adult sports participation

Rural older adult populations face significant disadvantages in sports participation compared to their urban counterparts, creating important public health and social equity concerns. Research consistently documents lower participation rates among rural older adults, with studies showing 15–30% lower engagement in organized physical activities compared to urban areas (19, 20). These disparities stem from multiple interconnected factors, including infrastructure limitations, resource scarcity, geographic isolation, and reduced social support networks (21, 22).

The infrastructure deficit in rural areas represents a fundamental barrier to older adult sports participation (23). Rural communities have significantly fewer recreational facilities per capita, with particularly pronounced shortages of age-appropriate exercise equipment and accessible indoor spaces for year-round activities (24). This infrastructure gap is compounded by transportation challenges, as rural older adults often face longer distances to reach available facilities and may lack reliable transportation options (25).

Resource constraints further exacerbate participation barriers in rural contexts. Rural communities typically operate with smaller budgets for recreational programming, fewer specialized staff, and limited access to professional expertise in older adult fitness and health promotion. These resource limitations create a cycle where reduced programming leads to lower participation, which in turn justifies continued underinvestment in older adult sports services.

However, recent research suggests that rural communities may possess unique assets that could support effective older adult sports participation when properly mobilized (26, 27). In the North American context, rural women, especially those in the southern United States, where individualistic family structures and market-based healthcare systems create different caregiving responsibilities compared to Chinese collective support systems, were more sedentary than urban women and reported more personal barriers to physical activity, citing caregiving duties as their top barrier (28). This pattern reflects the distinctive challenges of rural America's dispersed settlement patterns and limited public transportation infrastructure. Despite being more likely than urban residents to prefer and enjoy physical activity, rural residents in these Western contexts have fewer opportunities and receive less social support to engage in active pursuits. These barriers contrast with Chinese rural contexts where extended family systems and administrative village governance create different constraint patterns, as explored in our empirical analysis.

### 2.2 Public service delivery in rural contexts

The delivery of public services in rural areas presents distinct challenges that differentiate it from urban service provision. Rural governance systems must contend with smaller populations, limited economies of scale, geographic dispersion, and often more complex multi-jurisdictional arrangements (29, 30). These contextual factors create unique requirements for effective service delivery that standard urban-focused models may not adequately address.

Public service supply theory provides a foundational framework for understanding effective service delivery, emphasizing the integration of political commitment, institutional capacity, resource allocation, and implementation mechanisms (31, 32). However, applications of this theory to rural contexts remain limited, particularly regarding how these elements may interact differently in resource-constrained environments with distinctive governance structures (33).

Institutional theory offers additional insights into rural service delivery by highlighting the importance of formal and informal organizational arrangements (34, 35). Rural communities often rely more heavily on informal coordination mechanisms, volunteer networks, and hybrid public-private arrangements (36). These institutional variations may create alternative pathways to effective service delivery that differ substantially from formal bureaucratic models typically studied in urban contexts (37).

The complexity of rural governance arrangements suggests that effective service delivery may emerge through multiple alternative configurations rather than uniform approaches (38). This perspective aligns with contingency theory's emphasis on contextual adaptation and suggests that rural communities may develop locally-adapted solutions that achieve similar outcomes through different means (39, 40).

### 2.3 Configurational approaches in public administration

Traditional analytical approaches in public administration research have predominantly employed regression-based methods that assume uniform causal effects across contexts (41). These approaches may be inadequate for understanding complex public service outcomes that emerge through the interaction of multiple conditions in context-specific ways.

Qualitative Comparative Analysis (QCA) offers a methodologically sophisticated alternative that can accommodate the complex causation patterns characteristic of public service delivery (42). QCA's ability to identify multiple pathways to the same outcome (equifinality) and recognize that conditions may have different effects depending on context (conjunctural causation) makes it particularly suitable for analyzing rural governance systems where local contexts create diverse approaches to effectiveness (43, 44).

Necessary Condition Analysis (NCA) provides complementary insights by identifying conditions that must be present for desired outcomes to occur (12, 45). The integration of NCA and QCA enables a comprehensive analysis of both constraints (necessity) and opportunities (sufficiency) in public service delivery, offering a more nuanced understanding than either approach alone (14).

Recent applications of configurational methods in public administration have demonstrated their value for understanding complex policy outcomes, but applications to rural service delivery and older adult programming remain limited (46, 47). This represents a significant methodological gap given the apparent suitability of configurational approaches for analyzing the complex, context-dependent nature of rural governance (48).

### 2.4 Research gaps and theoretical integration

The literature review reveals three critical gaps that limit our understanding of effective rural older adult sports participation:

**Gap 1: Limited Configurational Analysis**—While extensive research documents barriers to rural older adult sports participation, few studies have examined how multiple conditions combine to create effective outcomes (49). The predominant focus on individual factors or simple interaction effects may miss important configurational patterns where conditions work together in complex ways (50).

**Gap 2: Necessity-Sufficiency Integration**—Existing research typically focuses on factors that correlate with better outcomes without distinguishing between conditions that are necessary vs. those that are sufficient (51). This distinction is crucial for policy development, as necessary conditions require universal attention while sufficient conditions offer alternative pathways (52).

**Gap 3: Equifinal Pathways**—The assumption that effective rural older adult sports participation requires similar approaches across communities may be incorrect (53). Different communities may achieve success through entirely different combinations of leadership, resources, and organizational arrangements, but this possibility has received little systematic investigation (54).

### 2.5 Empirical strategy and hypotheses

Based on the theoretical integration of public service supply theory, institutional theory, and contingency theory, this study develops a configurational model examining seven key conditions that may combine to produce effective older adult sports participation outcomes: Leadership Support (LD), Planning Systems (PS), Specialized Personnel (PN), Funding Allocation (FD), Facility Infrastructure (FT), Organizational Capacity (OG), and Activity Implementation (AT).

**Theoretical Model:** The model posits that effective outcomes emerge through configurations of conditions rather than simple additive effects. Different combinations of political commitment (Leadership Support), institutional capacity (Planning Systems and Organizational Capacity), resource allocation (Funding Allocation, Facility Infrastructure, and Specialized Personnel), and implementation mechanisms (Activity Implementation) may create alternative pathways to effectiveness.

#### 2.5.1 Hypothesis development

**Hypothesis H1: Necessity Hypothesis**—Based on the diversity of rural contexts and adaptive capacity of communities, no single condition will prove universally necessary for effective older adult sports participation. This hypothesis reflects the emphasis of contingency theory on multiple pathways to effectiveness and challenges assumptions about universal prerequisites.

**Hypothesis H2: Equifinality Hypothesis—**Multiple distinct sufficient configurations will exist, demonstrating that communities can achieve effective outcomes through different combinations of conditions. This hypothesis aligns with institutional theory's recognition of diverse organizational arrangements and public service supply theory's multi-dimensional framework.

**Hypothesis H3: Human Resource Centrality Hypothesis—**Specialized Personnel will appear across most sufficient configurations, reflecting the fundamental importance of dedicated human resources for translating policy intentions into practical outcomes. This hypothesis is grounded in resource-based theory and the concept of public service motivation.

**Hypothesis H4: Substitutability Hypothesis**—Leadership Support and resource-based conditions (Funding Allocation and Facility Infrastructure) will demonstrate substitutability patterns, where strong leadership can compensate for limited resources and adequate resources can enable effectiveness without strong leadership commitment. This hypothesis reflects research in public administration on alternative coordination mechanisms.

**Empirical Strategy—**The study employs integrated NCA–QCA analysis to test these hypotheses. The NCA examines whether any conditions prove necessary (H1), while the QCA identifies sufficient configurations (H2) and investigates the frequency patterns of conditions across configurations (H3 and H4). This methodological approach enables a comprehensive analysis of both constraints and opportunities in rural older adult sports participation.

## 3 Methodology

### 3.1 Research design and study population

This study examines village-level governance structures and public service provision mechanisms for older adult sports participation in rural China. Data were collected through the “Rural Older Adult(s) Public Sports Service Supply Status Survey” using a multi-stage stratified sampling approach to ensure geographic representativeness and variation in economic development levels.

Three regions representing China's distinct development patterns were selected: the Eastern region (Fuding City, Fujian Province), the Central region (Shaoyang County, Hunan Province), and the Western region (Guanghan City, Sichuan Province). Within each region, three townships were selected, representing high, medium, and low economic development levels based on per capita income indicators. The survey achieved comprehensive coverage through the complete enumeration of all villages within the nine selected townships, supplemented by additional villages from other townships within the three cities/counties. To enhance national representativeness, further data were collected during the 10th National “Village Leaders” Forum in Pengzhou, Sichuan Province.

The survey distributed 587 questionnaires to village leaders and officials, achieving 563 valid responses after removing 25 invalid cases (95.9% response rate). For the crisp-set QCA (csQCA) analysis, a refined subset of 156 villages was selected. This subset selection was necessary because csQCA requires complete case analysis without missing data, as the method relies on Boolean algebra to identify configurational patterns rather than statistical estimation techniques that can accommodate missing values.

### 3.2 Variable operationalization

**Outcome Variable:** Overall Sports Participation Rating (OC) was measured through village administrators' assessments of overall older adult sports participation in their villages, coded dichotomously as effective (1) for “excellent” or “good” evaluations vs. ineffective (0) for “average,” “poor,” or “very poor” evaluations.

**Condition Variables:** Seven key conditions were operationalized based on public service supply theory:

#### 3.2.1 Leadership and human resources

Leadership Support (LD) captures political commitment, coded as 1 when village leaders report older adult sports work as “quite important” or “very important.” This operationalization reflects the importance of local leadership commitment in driving public service initiatives in rural contexts.

Specialized Personnel (PN) reflects human resource investment, coded as 1 when villages have designated staff for older adult sports work. This operationalization recognizes that dedicated human resources are often necessary for the sustained implementation of the programs.

#### 3.2.2 Institutional capacity

Planning Systems (PS) measures institutional planning capacity through three components: work plans, special meetings, and assessment systems. The condition is coded as 1 when at least two elements are present, acknowledging that effective planning requires multiple institutional mechanisms working in coordination. This operationalization is based on institutional theory's emphasis on redundancy in organizational systems, where multiple planning mechanisms create institutional resilience while a single element may be insufficient for sustained coordination. Sensitivity analysis comparing 1/3 threshold (any element present) and 3/3 threshold (all elements required) demonstrated that the 2/3 threshold optimally balances inclusiveness with analytical rigor.

Organizational Capacity (OG) measures organizational structure through coordinators and sports organizations, coded as 1 when villages have ≥2 coordinators AND ≥1 organization. This reflects the conjunctural nature of organizational requirements, where both human coordination capacity and institutional structures are needed.

#### 3.2.3 Resource allocation

Funding Allocation (FD) represents financial resource commitment, coded as 1 when villages allocate specific budgets for older adult sports activities. This condition captures the importance of dedicated financial resources in enabling program sustainability.

Facility Infrastructure (FT) combines physical infrastructure elements, including activity centers, specialized facilities, and exercise sites. The condition is coded as 1 when villages have centers/facilities AND at least one exercise site, reflecting the conjunctural nature of infrastructure requirements. The conjunctural coding reflects the complementary nature of formal and informal physical infrastructure. Activity centers provide Organizational Capacity while exercise sites enable actual participation, aligning with research showing that indoor organizational spaces and outdoor activity areas work synergistically. Sensitivity analysis testing these two components separately showed that the conjunctural condition has higher predictive consistency than either component alone.

#### 3.2.4 Implementation mechanisms

Activity Implementation (AT) captures service delivery frequency by combining organized activities, health education, and promotional campaigns. The condition is coded as 1 when total annual activities ≥6, establishing a threshold that reflects meaningful program activity levels. The six-activity threshold approximates bi-monthly programming, representing a minimum frequency for sustained engagement, as outlined in community participation literature. Alternative thresholds (4, 8, 10 activities) were tested, showing that the 6-activity threshold achieves optimal balance between covering meaningful program activity levels and avoiding overly restrictive standards. Thresholds below 6 included sporadic activities potentially insufficient for sustained older adult engagement, while thresholds above 6 excluded programs that operate effectively under resource constraints. Detailed questionnaire items are provided in Supplementary Table S1.

### 3.3 Analytical strategy

All analyses were conducted in R version 4.3.0 using the NCA package (version 3.1.2) for necessity analysis and the QCA package (version 3.22) for qualitative comparative analysis.

Necessary Condition Analysis (NCA): Following Dul's (12) protocols, NCA identified conditions that must be present for effective outcomes (55). The analysis employed ceiling envelopment with free disposal hull (CE-FDH) and ceiling regression with free disposal hull (CR-FDH) techniques with 10,000-iteration permutation tests. Conditions were identified as necessary when exhibiting effect sizes (*d*) >0.1, significance (*p* < 0.05), and accuracy > 95% (56). Effect sizes were interpreted using established benchmarks: small (0.1 ≤ *d* < 0.3), medium (0.3 ≤ *d* < 0.5), and large (*d* ≥ 0.5).

Qualitative Comparative Analysis (QCA): Following Ragin's (10) framework, QCA identified sufficient condition combinations. Truth tables were constructed with an inclusion cut-off at 0.8, a frequency cut-off of 1, and a PRI cut-off at 0.7. Three solution types were generated: complex, parsimonious, and intermediate solutions (57). This study employed the intermediate solution to construct the configurational table, as it represents the most theoretically meaningful and practically interpretable pathway among the three solution types (58). The intermediate solution strikes an optimal balance between parsimony and complexity by incorporating substantive theoretical knowledge while avoiding both the overly restrictive assumptions of the parsimonious solution and the excessive complexity of the complex solution. This approach enables the identification of core conditions that are consistently present across multiple pathways while maintaining theoretical coherence with public service supply theory (59). Solution quality was assessed using consistency (≥0.80), raw coverage, and unique coverage measures. Sensitivity analysis examined alternative thresholds (0.75–0.85) to validate stability.

Integration Strategy: NCA and QCA results were integrated sequentially, with NCA identifying necessary foundational requirements and QCA revealing sufficient combinations for success (60). This approach provides comprehensive insights into both constraints (necessity) and opportunities (sufficiency) in rural older adult sports participation.

## 4 Results

### 4.1 Necessary condition analysis results

The Necessary Condition Analysis (NCA) examined whether any of the seven conditions must be present for effective older adult sports participation outcomes. Table 1 presents the results using both ceiling envelopment with free disposal hull (CE-FDH) and ceiling regression with free disposal hull (CR-FDH) techniques.

**Table 1:** Necessary condition analysis results.

**Condition**	**Method**	**c-Accuracy (%)**	**Ceiling zone**	**Scope**	**Effect size**	***p*-value**
LD	CE-FDH	100	0	1	0	1
	CR-FDH	100	0	1	0	1
PN	CE-FDH	100	0	1	0	1
	CR-FDH	100	0	1	0	1
PS	CE-FDH	100	0	1	0	1
	CR-FDH	100	0	1	0	1
FD	CE-FDH	100	0	1	0	1
	CR-FDH	100	0	1	0	1
FT	CE-FDH	100	0	1	0	1
	CR-FDH	100	0	1	0	1
OG	CE-FDH	100	0	1	0	1
	CR-FDH	100	0	1	0	1
AT	CE-FDH	100	0	1	0	1
	CR-FDH	100	0	1	0	1

LD, Leadership Support; PN, Specialized Personnel; PS, Planning Systems; FD, Funding Allocation; FT, Facility Infrastructure; OG, Organizational Capacity; AT, Activity Implementation.

The analysis reveals that none of the seven conditions meet the established criteria for necessity. All conditions demonstrate effect sizes (*d*) of 0, with ceiling zones of 0 and non-significant *p*-values (*p* = 1), consistent across both analytical techniques.

The absence of necessary conditions indicates that effective rural older adult sports participation does not depend on any single, universally required condition. This suggests that villages can achieve success through multiple alternative pathways without requiring specific baseline conditions to be present in all cases.

This finding aligns with the configurational nature of rural governance, where communities with different resource endowments and organizational structures may succeed through entirely different approaches. Rather than identifying universal prerequisites, the results highlight the importance of examining how conditions work in combination, setting the foundation for the subsequent QCA analysis of sufficient condition configurations.

### 4.2 Qualitative comparative analysis results

Following the Necessary Condition Analysis, QCA was employed to identify sufficient combinations of conditions that lead to effective older adult sports participation. Since all variables were operationalized as binary (0/1) indicators, this study utilized crisp-set QCA (csQCA), which does not require calibration procedures as the data are already in a dichotomous format suitable for Boolean analysis. The intermediate solution generated six distinct configurational pathways (S1–S6) that achieve the outcome, with an overall solution consistency of 0.886 and solution coverage of 0.43.

**Figure 1:**
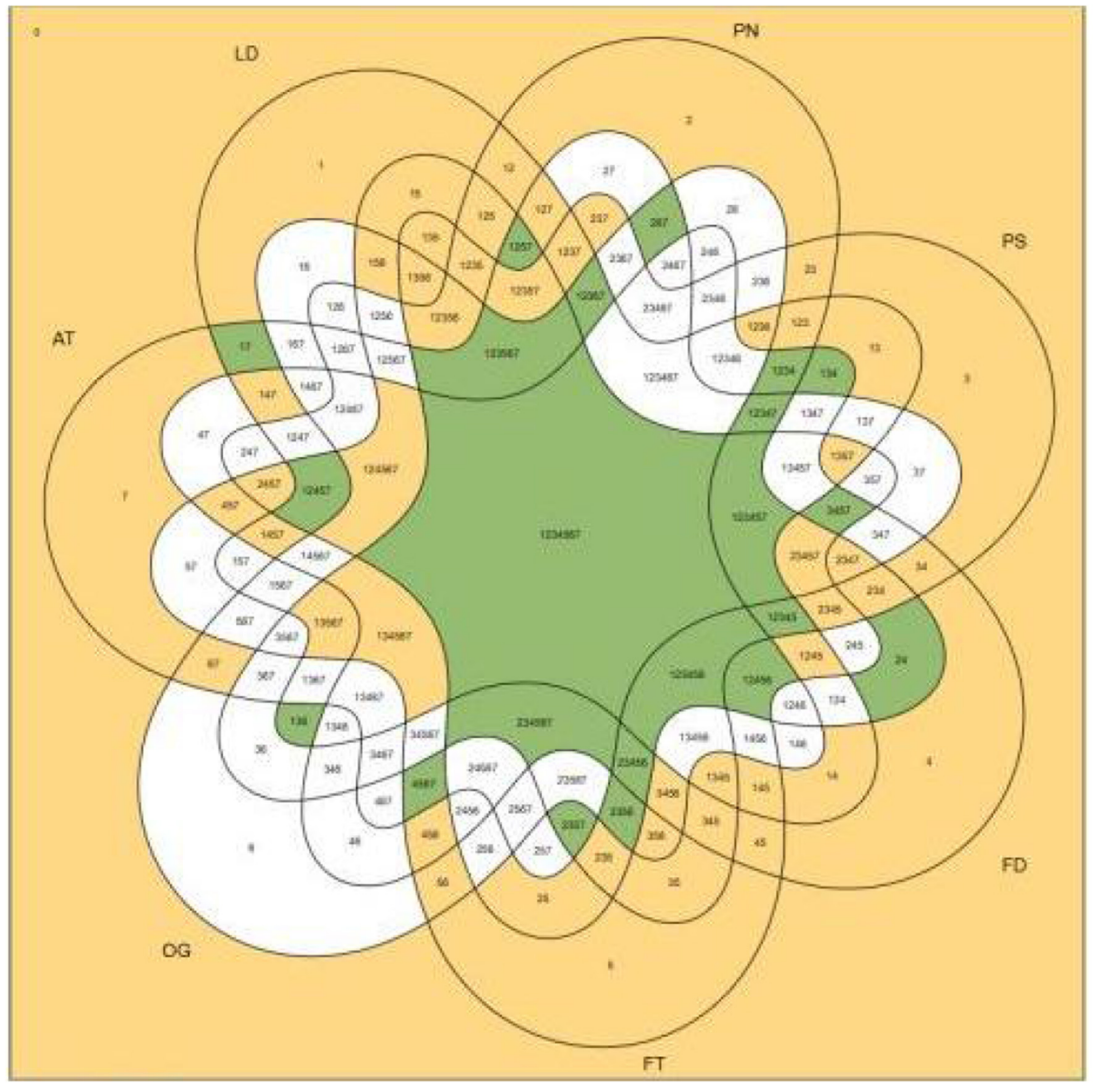
Venn diagram of condition combinations across 156 villages.

The analysis constructed a truth table encompassing all possible combinations of the seven conditions. Figure 1 illustrates the complex overlapping patterns of these conditions across the 156 villages, demonstrating the configurational diversity underlying rural older adult sports participation. After applying the inclusion cut-off threshold of 0.8, frequency cut-off of 1, and PRI consistency cut-off of 0.7, the logical minimization process identified six sufficient pathways. The solution demonstrates equifinality, where multiple distinct combinations of conditions can lead to the same outcome, reflecting the complex and context-dependent nature of rural governance. The specific results are presented in Table 2.

**Table 2:** Main configurations for effective rural older adult sports participation.

**Configuration**	**Solutions**					
	**S1**	**S2**	**S3**	**S4**	**S5**	**S6**
LD	•	•	•	•	•	
PN	•	•	•	•		•
PS	•	•			•	•
FD	○	•	•		•	•
FT				•	•	•
OG		○	○	○	○	•
AT	•			•	○	•
covS	0.064	0.209	0.123	0.081	0.123	0.085
covU	0.051	0.043	0.047	0.013	0.013	0.009
inclS	0.882	0.86	0.935	0.864	0.853	0.952
solCons	0.886					
solCov	0.43					

• , condition present; blank, condition absent; ○, don't care condition (condition can be either present or absent). LD, Leadership Support; PN, Specialized Personnel; PS, Planning Systems; FD, Funding Allocation; FT, Facility Infrastructure; OG, Organizational Capacity; AT, Activity Implementation; covS, solution coverage; covU, unique coverage; inclS, solution consistency; solCons, overall solution consistency; solCov, overall solution coverage.

For the configurational analysis, the PRI consistency threshold was set at 0.7, case frequency at 1, and raw consistency threshold at 0.8.

**Solution 1 (Leadership-Governance Model):** LD•PN•PS•AT (covS = 0.064, covU = 0.051, inclS = 0.882). This configuration represents a governance-oriented approach emphasizing leadership commitment (LD), dedicated personnel (PN), institutional Planning Systems (PS), and active programming (AT). The presence of funding as a “don't care” condition (FD) indicates that villages can achieve effectiveness through this pathway regardless of formal budget allocation. This suggests that strong governance structures can compensate for limited financial resources through effective coordination and volunteer-based implementation.

**Solution 2 (Resource-Intensive Leadership Model):** LD•PN•PS•FD (covS = 0.209, covU = 0.043, inclS = 0.86). This pathway demonstrates the highest raw coverage (0.209), indicating its empirical importance in explaining effective cases. It combines Leadership Support with institutional planning and dedicated funding, suggesting a more formalized approach to service delivery. The “don't care” status of organizational construction (OG) implies that formal organizations may not be essential when strong leadership and adequate resources are present.

**Solution 3 (Streamlined Resource Model):** LD•PN•FD (covS = 0.123, covU = 0.047, inclS = 0.935). This solution achieves high consistency (0.935) with a relatively parsimonious combination of leadership, personnel, and funding. The absence of Planning Systems and infrastructure requirements suggests that some villages achieve effectiveness through flexible, resource-backed approaches without formal institutional frameworks. This pathway may be particularly relevant for smaller communities with strong informal coordination mechanisms.

**Solution 4 (Infrastructure Activity Model):** LD•PN•FT•AT (covS = 0.081, covU = 0.013, inclS = 0.864). This configuration emphasizes the combination of leadership, personnel, physical facilities, and active programming. The “don't care” status of organizational construction (OG) suggests that formal organizations are not essential when strong infrastructure and programming are present. This pathway may represent communities that have successfully developed physical assets and maintain high activity levels through direct leadership involvement.

**Solution 5 (Comprehensive Integration Model):** LD•PS•FD•FT•AT (covS = 0.123, covU = 0.013, inclS = 0.853). This represents the most comprehensive pathway, integrating institutional planning, financial resources, physical infrastructure, and active programming under strong leadership. The “don't care” status of organizational construction (OG) indicates that formal organizational structures may be less critical when multiple other supportive elements are aligned. This pathway suggests a holistic approach that may be more feasible for resource-rich communities.

**Solution 6 (Organizational Excellence Model):** PN•PS•FD•FT•OG•AT (covS = 0.085, covU = 0.009, inclS = 0.952). This solution achieves the highest consistency (0.952) and represents the only pathway that does not require Leadership Support, instead relying on organizational excellence through comprehensive institutional, resource, and operational integration. The high consistency suggests that when all other elements are optimally aligned, formal leadership commitment becomes dispensable, indicating that well-established organizational systems can operate effectively through institutionalized processes.

#### 4.2.1 Core and peripheral conditions analysis

Core Conditions: Leadership Support (LD) emerges as the most critical condition, appearing in five of six solutions (83.3% frequency), establishing it as a core element across most pathways to effectiveness. Specialized Personnel (PN) demonstrates equally high importance, present in all six solutions (100% frequency), indicating that dedicated human resources represent a universal element across all effective configurations.

Frequent Conditions: Planning Systems (PS) and Funding Investment (FD) each appear in four solutions (66.7% frequency), suggesting their substantial but not universal importance. Activity Implementation (AT) appears in four solutions, indicating the significance of active programming.

Conditional Importance: Facility Infrastructure (FT) and Organizational Construction (OG) each appear in three solutions (50% frequency), suggesting their importance in specific configurational contexts rather than as universal requirements.

The frequent appearance of “don't care” conditions across solutions provides insights into conditional flexibility. Organizational Construction (OG) appears as “don't care” in four solutions, suggesting that formal organizational structures may be substitutable with other coordination mechanisms. This finding indicates that villages can achieve effectiveness through either formal organizational development or alternative coordination approaches. The overall solution coverage of 0.43 indicates that these six pathways collectively explain 43% of effective cases, suggesting that while substantial, additional pathways likely exist beyond those identified. The solution consistency of 0.886 demonstrates that these configurations reliably lead to effective outcomes in approximately 89% of cases, indicating strong causal relationships. The unique coverage values reveal each pathway's distinctive contribution: Solution 1 (covU = 0.051) and Solution 3 (covU = 0.047) demonstrate the highest unique contributions, suggesting that these represent particularly distinct approaches to achieving effectiveness.

### 4.3 Robustness assessment

To explore the robustness of the results, two methods were employed following established QCA protocols (55, 56). First, the consistency threshold was increased from 0.8 to 0.85, with the adjusted results retaining four of the original six configurations (S2, S3, S5, and S6), demonstrating stability under more stringent criteria. Second, the PRI threshold was increased from 0.7 to 0.75, yielding three robust configurations (S3, S5, and S6) that represented a subset of the original solutions. Both tests confirmed that the identified pathways represent stable causal relationships rather than methodological artifacts, with Leadership Support and Specialized Personnel maintaining their status as core conditions across all specifications. This indicates that the study results passed the robustness test and are methodologically reliable.

## 5 Discussion

Activity Implementation (AT) captures service delivery frequency by combining organized activities, health education, and promotional campaigns (61). The condition is coded as 1 when total annual activities ≥6, establishing a threshold that reflects meaningful program activity levels. The six-activity threshold approximates bi-monthly programming, representing a minimum frequency for sustained engagement based on community participation literature (62). Alternative thresholds (4, 8, 10 activities) were tested, showing that the 6-activity threshold achieves optimal balance between covering meaningful program activity levels and avoiding overly restrictive standards. Thresholds below 6 included sporadic activities potentially insufficient for sustained older adult engagement, while thresholds above 6 excluded programs that operate effectively under resource constraints. The absence of necessary conditions, as revealed through NCA, appears consistent with recent configurational research indicating that complex social outcomes may rarely depend on single, universally required factors (63). This finding resonates with Fiss's (53) seminal work on organizational configurations, which emphasizes that successful outcomes emerge through multiple pathways rather than uniform prerequisites (53). The identification of six distinct sufficient pathways through QCA substantiates the principle of equifinality in rural governance systems, supporting Greckhamer et al.'s (48) argument that complex social phenomena exhibit causal heterogeneity, where different combinations of conditions can produce identical outcomes (48). The configurational approach reveals that rural communities can achieve effective older adult sports participation through fundamentally different strategic orientations, from resource-intensive models (Solution 2) to governance-focused approaches (Solution 1), reflecting what Meyer et al. (40) characterized as organizational fit through alternative configurations.

The emergence of Leadership Support (LD) as a core condition appearing in five of six pathways provides empirical support for transformational leadership theory in public service contexts, aligning with research by Tummers and Knies (64), who demonstrated that public leadership significantly influences service delivery effectiveness through its impact on organizational climate and employee motivation (64). However, the existence of Solution 6 (Organizational Excellence Model), which achieves effectiveness without Leadership Support, suggests that institutionalized organizational systems can sometimes substitute for individual leadership, supporting institutional theory's emphasis on embedded routines and structures (34). The universal presence of Specialized Personnel (PN) across all six configurations represents one of this study's most significant theoretical contributions, extending public service motivation theory by demonstrating that dedicated human resources constitute an invariant element in effective rural service delivery (65). The consistency of this finding across diverse configurational contexts supports Vandenabeele's (66) proposition that specialized public service capacity represents a fundamental requirement for translating policy intentions into practical outcomes. This human resource centrality provides empirical validation for resource-based view applications in public administration, where specialized capabilities create sustainable competitive advantages (67).

These configurational patterns must be understood within the broader context of fundamental barriers constraining rural older adult sports participation in China. Rural villages face interconnected structural challenges that traditional linear approaches have struggled to address comprehensively. Economic resource constraints represent the most visible barrier, with rural villages typically operating on limited budgets where basic infrastructure and social services compete for scarce funding (68). This scarcity explains why FD appears in multiple pathways yet remains substitutable; villages develop creative financing mechanisms through resource sharing, volunteer coordination, and utilization of multi-purpose facility. Demographic transformation poses deeper challenges, as youth outmigration has created aging populations with diminished social capital and reduced volunteer capacity (69, 70). This demographic hollowing-out directly explains why PN emerges as universal across configurations—dedicated human resources become critical when natural support networks erode. Geographic isolation and infrastructure limitations further compound these challenges, creating transportation barriers and facility accessibility issues that disproportionately affect older adult populations (71, 72). However, our configurational analysis reveals that these barriers operate synergistically rather than independently, with different villages experiencing distinct combinations requiring tailored interventions rather than uniform solutions.

The frequent appearance of “don't care” conditions across solutions reveals important insights about adaptive governance mechanisms in rural contexts. Organizational Construction (OG) appearing as “don't care” in four solutions suggests that formal organizational structures may be less critical than previously assumed, supporting arguments for flexible governance arrangements that can adapt to local contexts (73). This finding aligns with Ostrom's (74) work on institutional diversity, which emphasizes the importance of locally adapted governance mechanisms over standardized organizational forms. The substitutability patterns observed across configurations provide empirical support for the application of contingency theory in public administration, demonstrating that different combinations of resources and capabilities can produce equivalent outcomes (39). The configurational analysis reveals sophisticated patterns of resource complementarity and strategic trade-offs that extend beyond simple additive relationships. Solution2's high raw coverage (0.209) combined with comprehensive resource integration suggests that certain combinations create synergistic effects, supporting arguments for holistic rather than piecemeal approaches to rural development (75). This finding resonates with research by Elbanna et al. (2016) on public service improvement, which demonstrates that simultaneous investments across multiple dimensions often produce superior outcomes compared to sequential improvements (76).

The study's findings shed light on the complex mechanisms underlying rural–urban disparities in older adult sports participation. The configurational pathways reveal that rural communities face fundamentally different challenges compared to urban areas, where standardized service delivery models may be more feasible (77). The necessity for multiple alternative pathways in rural contexts reflects what Rodríguez-Pose (78) characterized as place-based development requirements, where local context fundamentally shapes effective intervention strategies. The absence of universally necessary conditions challenges centralized policy approaches that assume uniform implementation requirements across diverse rural contexts, supporting arguments for decentralized governance models that allow local communities to pursue context-appropriate strategies while maintaining accountability for outcomes (79). The varying consistency levels across solutions (ranging from 0.853 to 0.952) provide insights into the relative reliability of different strategic approaches, with Solution6′s exceptional consistency (0.952) suggesting that comprehensive organizational development may represent the most reliable pathway to effectiveness, although its low unique coverage (0.009) indicates limited empirical prevalence (80).

The successful integration of NCA and QCA methodologies contributes to the growing literature on mixed-method configurational analysis, with the complementary insights from necessity and sufficiency analyses demonstrating the value of methodological pluralism in understanding complex social phenomena (81). The finding that no conditions prove necessary while multiple sufficient configurations exist illustrates what Vis and Dul (14) described as the distinctive contributions of these complementary approaches (82). This methodological approach addresses recent calls for more sophisticated analytical techniques in public administration research, moving beyond traditional variable-oriented approaches to reveal complex causal mechanisms underlying effective service delivery (83). However, several limitations constrain the generalizability and interpretation of these findings. The focus on rural Chinese contexts limits direct applicability to other national settings, although the configurational principles may translate across similar developmental contexts (84). The cross-sectional design prevents examination of temporal dynamics and pathway evolution, representing an important avenue for future longitudinal research (85). The solution coverage of 0.43 indicates that additional pathways likely exist beyond those identified, suggesting opportunities for further configurational exploration with larger samples or different analytical parameters (42).

The Chinese rural context presents unique characteristics that distinguish these findings from Western rural development experiences. China's rapid economic transformation has created stark development disparities between regions, with eastern coastal villages often possessing resources comparable to those of urban areas, while western mountainous villages remain severely constrained (86). This heterogeneity explains the configurational diversity observed in our analysis—what works in resource-rich eastern villages may be entirely inappropriate for resource-constrained western communities. Additionally, China's administrative village system creates formal governance structures that facilitate certain pathways (particularly Solutions 2 and 5, emphasizing Planning Systems and formal funding) while traditional social networks enable alternative approaches (Solutions 1 and 3 relying on informal coordination) (87). The Confucian emphasis on collective responsibility and respect for older adult populations provides cultural foundations for community-based sports programming. Hitherto, this cultural capital varies significantly across regions and generational cohorts (88). Understanding these contextual specificities is crucial for interpreting the practical applicability of our configurational findings beyond the Chinese setting.

The configurational findings offer practical guidance for rural development practitioners and policymakers. The identification of multiple pathways suggests that communities should conduct comprehensive assessments of their existing capabilities and resources before selecting development strategies. The centrality of Specialized Personnel across all pathways indicates that human resource development should receive priority attention regardless of the chosen strategic orientation (89). The configurational flexibility revealed by this study suggests that rural development programs should offer multiple implementation options rather than prescriptive approaches, supporting arguments for adaptive management strategies that allow communities to experiment with different combinations based on local conditions and capabilities (90). The success of diverse pathways demonstrates that effective rural development requires embracing local diversity rather than pursuing standardization (91). Future research could examine the temporal stability of these configurations and investigate the mechanisms through which communities transition between pathways, while incorporating fuzzy-set approaches to reveal more nuanced relationships among partially developed conditions.

## 6 Conclusion

This study employed Necessary Condition Analysis (NCA) and Qualitative Comparative Analysis (QCA) to examine the configurational pathways underlying effective rural older adult sports participation in China. Through analysis of data from 156 villages across three distinct regional contexts, our findings contribute to understanding the complex causation mechanisms that drive successful public service delivery in rural settings.

The NCA results indicate that no single condition serves as universally necessary for effective older adult sports participation, suggesting that rural communities can achieve success without requiring specific baseline prerequisites. This absence of necessary conditions reflects the adaptive nature of rural governance systems, where communities with diverse resource endowments and organizational capabilities may pursue entirely different strategic approaches. The finding challenges policy frameworks that assume uniform implementation requirements and supports arguments for more flexible, context-responsive governance models.

The QCA analysis identified six distinct configurational pathways to effectiveness, demonstrating the principle of equifinality in rural public service delivery. These pathways range from governance-focused approaches (Solution 1), which emphasize leadership and institutional coordination, to resource-intensive models (Solution 2), which integrate formal planning and dedicated funding. The emergence of an Organizational Excellence Model (Solution 6) that operates effectively without traditional Leadership Support suggests that well-established institutional systems can sometimes substitute for individual leadership commitment. This configurational diversity indicates that effective rural development strategies should accommodate multiple alternative approaches rather than prescribing standardized solutions. Several key insights emerge from the configurational analysis. Specialized Personnel appears as the only condition present across all pathways, suggesting that dedicated human resources may represent a fundamental requirement for translating policy intentions into practical outcomes. Leadership Support demonstrates high frequency across configurations, reinforcing its importance while acknowledging that alternative coordination mechanisms may sometimes suffice. The frequent appearance of “don't care” conditions, particularly for Organizational Construction, reveals significant substitutability among different governance arrangements, indicating that formal organizational structures may be less critical than previously assumed when other supportive elements are aligned.

The methodological integration of NCA and QCA provides complementary insights into both constraints and opportunities in rural governance. While NCA reveals the absence of universal prerequisites, QCA illuminates the multiple sufficient pathways available to communities. This dual perspective addresses recent calls for more sophisticated analytical approaches in public administration research and demonstrates the value of configurational methods for understanding complex social outcomes. The findings offer several practical implications for rural development policy and practice. The identification of multiple pathways suggests that communities should conduct comprehensive assessments of their existing capabilities before selecting development strategies. The centrality of Specialized Personnel across all configurations indicates that human resource development deserves priority attention regardless of chosen strategic orientation. The observed configurational flexibility suggests that rural development programs should offer multiple implementation options rather than prescriptive approaches, enabling communities to experiment with different combinations based on local conditions and capabilities.

However, several limitations constrain the generalizability of these findings. The focus on rural Chinese contexts may limit the direct applicability to other national settings, although the configurational principles may translate across similar developmental environments. The cross-sectional design prevents examination of temporal dynamics and pathway evolution, representing an important avenue for future research. The solution coverage of 0.43 indicates that additional pathways likely exist beyond those identified, suggesting opportunities for further configurational exploration with larger samples or alternative analytical parameters.

## Data Availability

The raw data supporting the conclusions of this article will be made available by the authors, without undue reservation.
