# Supplementary material for: Configurational pathways to effective rural older adult sports participation: a necessity and sufficiency analysis using NCA and QCA
Source: Front Public Health. 2025 Oct 27;13:1695787. doi: 10.3389/fpubh.2025.1695787 (PMC12604643; doi:10.3389/fpubh.2025.1695787)
Supplement: Supplementary file 1 [file Data_Sheet_1.docx]

**Supplementary Materials**

**Supplementary Material S1: Complete R Code for NCA and QCA Analysis**

This supplementary file contains the complete R code used for both Necessary Condition Analysis (NCA) and Qualitative Comparative Analysis (QCA) in this study. The code is organized into the following sections:

Data Preparation and Loading: Code for importing and preprocessing the village-level survey data, including variable coding and transformation procedures.

Necessary Condition Analysis (NCA): Complete R code using the NCA package (version 3.1.2) implementing both CE-FDH and CR-FDH techniques with 10,000-iteration permutation tests for all seven conditions.

Qualitative Comparative Analysis (QCA): R code using the QCA package (version 3.22) for crisp-set analysis, including:

Truth table construction with specified thresholds (inclusion cut-off = 0.8, frequency cut-off = 1, PRI cut-off = 0.7)

Logical minimization procedures

Generation of complex, parsimonious, and intermediate solutions

Robustness Testing: Code for sensitivity analyses examining alternative consistency thresholds (0.75-0.85) and PRI thresholds to validate solution stability.

Data Visualization: Code for generating the seven-way Venn diagram (Figure 1) and other analytical visualizations.

Results Export: Functions for extracting and formatting results tables, including consistency and coverage measures.

The code includes detailed comments explaining each analytical step and can be used to reproduce all results presented in the main manuscript. All variable names correspond to those defined in the methodology section: LD (Leadership Support), PN (Specialized Personnel), PS (Planning Systems), FD (Funding Allocation), FT (Facility Infrastructure), OG (Organizational Capacity), AT (Activity Implementation), and OC (Overall Sports Participation Rating).

# 二、载入必要的程序包

install.packages(c("QCA", "SetMethods", "admisc"),

dependencies = TRUE)

library(QCA)

library(SetMethods)

library(admisc)

library(ggrepel)

library(ggplot2)

# 三、导入原始数据

qca <- read.csv("示例数据.csv", row.names = 1, fileEncoding = "GBK")

# 四、校准

# （二）使用循环为每个变量应用校准

for (var in names(calibrations)) {

qca[[var]] <- calibrate(qca[[var]], type = "fuzzy", method = "direct", thresholds = calibrations[[var]])

}

# （三）导出校准后的数据

write.csv(qca, "校准后.csv", fileEncoding = "GBK")

# （四）读取校准后数据

qca <- read.csv("校准后.csv", row.names = 1, fileEncoding = "GBK")

# 五、单个条件的必要性分析

# （一）选取条件变量

conditions <- qca[, 1:6]

# （二）必要性分析

pof(conditions, Y, data = qca)

pof(1-conditions, Y, data = qca)

pof(conditions, ~Y, data = qca)

pof(1-conditions, ~Y, data = qca)

# （三）绘制散点图

XYplot(X1, Y, data = qca, jitter = TRUE, clabels = rownames(qca))

XYplot(X1, Y, data = qca, jitter = TRUE, clabels = seq(nrow(qca)))

# 六、条件组态的充分性分析（组态分析）

# （一）构建真值表

zzb <- truthTable(qca, outcome = "Y", conditions = "X1,X2,X3,X4,X5,X6,x7",

incl.cut = 0.8, n.cut = 1, pri.cut = 0.7,

show.cases = TRUE, sort.by = "OUT", complete = TRUE)

zzb

# （二）寻找必要的组合，ron.cut代表必要相关性，参考标准为0.6

sp <- superSubset(qca, outcome = "Y", conditions = "X1,X2,X3,X4,X5,X6,x7",

incl.cut = 0.90, ron.cut = 0.5)

sp

# （三）寻找矛盾的逻辑余项

findRows(obj = zzb, type = 2)

# （四）寻找一致性异常的案例

zzb1 <- truthTable(qca, outcome = "Y", conditions = "X1,X2,X3,X4,X5,X6,x7",

incl.cut = 0.8, n.cut = 1, pri.cut = 0.7,

show.cases = TRUE, sort.by = "OUT", complete = TRUE, dcc = TRUE)

zzb1

# （五）清洗真值表

newzzb <- esa(oldtt = zzb, nec_cond = c("X3+~X4+X6"),

untenable_LR = c("X1*X2*X3*~X4*~X5*~X6"))

# （六）查看新的真值表

newzzb

# （七）求（增强版）复杂解

# 1.查看复杂解

c <- minimize(newzzb, details = TRUE)

c

# 2.查看复杂解所有的质蕴含项

c$PIchart

# （八）求增强版简单解

# 1.查看简单解

p <- minimize(newzzb, include = "?", details = TRUE)

p

# 2.查看简单解所有的质蕴含项

p$PIchart

# 3.查看所有有贡献的逻辑余项

p$SA

# （九）求增强版中间解

# 1.查看中间解

i <- minimize(newzzb, include = "?", dir.exp= "1,1,1,1,1,1,1", details = TRUE)

i

# 2.寻找符合方向预期的逻辑余项（容易的反事实）

i$i.sol$C1P1$EC

# 3.寻找不符合方向预期的逻辑余项（困难的反事实）

i$i.sol$C1P1$DC

# 4.查看所有质蕴含项

i$i.sol$C1P1$PIchart

# （十）绘制典型案例散点图

# 1.计算组态的隶属度

sol <- compute(X1*X3*~X4*X5*~X6, data = qca)

# 2.设置只显示典型案例

col <- rep("black", nrow(qca))

col[sol < 0.5 | qca$Y < 0.5] <- "NA"

clabels <- logical(nrow(qca))

clabels[sol > 0.5 & qca$Y > 0.5] <- TRUE

# 3.绘制散点图

XYplot(sol, Y, data = qca,

jitter = TRUE, clabels = clabels, model = TRUE,

cex = 0.7,

col = col, bg = col, xlab = "X1*X3*~X4*X5*~X6")

# （十一） 绘制维恩图

runGUI()

# 0.安装NCA包，如已安装请忽略

install.packages("NCA")

# 1.运行NCA包

library(NCA)

# 2.设置工作目录

setwd("C:/Users/ZhuanZ/Desktop/读个博士吧，求求了/老年体育供给原始数据")

# 3.读取数据

jiafei <- read.csv("QCA_data.csv", fileEncoding = "GBK")

# 4.运行NCA分析（d＞0.1，P＜0.05，精确度＞95%，就可以判定为是必要条件）

model <- nca_analysis(jiafei, c(2:8), 1, test.rep=10000, flip.x = c(TRUE,TRUE,TRUE,TRUE,TRUE,TRUE,TRUE), flip.y = TRUE)

nca_output(model, test = TRUE)

# 2. 尝试不同的flip设置

model2 <- nca_analysis(jiafei, c(2:8), 1, test.rep=10000, flip.x = FALSE, flip.y = FALSE)

nca_output(model2)

# 查看CR方法的瓶颈表

model2$bottlenecks$cr_fdh
